# Prescriptions and Formulæ

**Published:** 1877-03-20

**Authors:** 


### PRESCRIPTIONS AND FORMULÆ

*Strumous Ophthalmia.*—

R. Hydrargyri oxidi flavi....gr.vi,
Unguenti simplicis..........dr.j.

A piece of the size of a hempseed to be inserted between the eyelids every night.

R. Hydrargyri perchloridi....gr.ij.
Ammoni chloridi..........gr.iij.
Aquæ......................ad.oz.ij. S.

One teaspoonful to be mixed with half a small teacupful of lukewarm water, and used as a lotion for the eyes every four hours. In bathing the eyes care should be taken that the lotion is applied inside, and the eyelids dried thoroughly after each application of the lotion. The eyes must be kept covered by means of a light bandage. When the inflammation subsides the bandage should be removed, but the lotion to be continued for three or four weeks to assist in removing the opacitie. Cods-liver oil and syrup of the iodide of iron are to be given for a lengthened period. —*Brit. Med. Jonr.*

*Improued Cathartic Pills:*—

Compound ext. colocynth...24 grs.
Ext. jalap.......................12 "
Resin of podophyllum........ 6 "
Ext. leptandra......... .....12 "
Extract of hyosciamus.......12 "
Oil of pepperment............. 6 drops.

To be divided into twenty-four pills. Dose the same as the ordinary anti-bilious pills. They do not gripe.—*Druggists' Circular.*

*For Asthmatic Paroxysm.*—

R. Ether.....................fl.oz.iss.
Tinct. lobelia...............fl.oz.j.
Tinct. opii.................fl.oz.ss.
Tinct. stramon ..........fl.oz.ss.

M. Dose, a teaspoonful every one or two hours, until nausea is produced.

*LaFayette Mixture.*—

Balsam of copaiba...... 1 fluid ounce.
Solution of potassa..... 2 " drachms.
Sweet spirit of nitre.... 1 " ounce
Syrup of gum........... 6 " ounces.
Oil of wintergreen......16 drops.

Some add four drachms of powdered extract of liquorice, or four fluid drachchms of the fluid extract. The mixture is best made by first shaking in the bottle the balsam with the potassa and the sweet spirit of nitre, and then adding the other ingredients. The dose is a tablespoonful three times a day after meals.—*Druggists' Circular.*

*Emulsion of Phosphorus.*—

R. Phosphori......................gr.j.
Chloroform pur..........fl.dr.ij.

M. Dissolve by shaking together in a bottle· Add

Ol gaultheriæ............fl.dr.ss.
Spts. vini gallici...... .fl.dr.iij.
Syr. acaciæ..............fl.oz.vij.

M. Ft. emulsio. Each teaspoonful contains one-sixtieth of a grain of phosphorus.

*Alterative and Tonic for Chronic Pharyngitis*—

R. Potass. iodid..............oz.ss.
Tinct. rhei................fl.oz.ij.
Syr. sarsaparillæ comp.fl.oz.iv.

M. S. Dose, a teaspoonful in water after each meal. —*Louisville Medical News.*

*For Asthma in Intervals.*—

R. Potass. iodid..............dr.iss.
Spts. ammon. arom .....fl.oz.j.
Tinct. belladon..........fl.oz.ss.
Tinct. cinch. comp .....fl.oz.ij.
Aquæ menth. pip.....fl.oz.ivss.

M. S. Dose, a tablespoonful after each meal.

